# Posttraumatic Growth after the Fukushima Nuclear Disaster: Examination of Free Descriptions among Fukushima Residents Who Lived in the Evacuation Area

**DOI:** 10.3390/ijerph19010192

**Published:** 2021-12-24

**Authors:** Hajime Iwasa, Chihiro Nakayama, Nobuaki Moriyama, Masatsugu Orui, Seiji Yasumura

**Affiliations:** 1 Department of Public Health, School of Medicine, Fukushima Medical University, Fukushima 960-1295, Japan; nakac@fmu.ac.jp (C.N.); moriyama@fmu.ac.jp (N.M.); oruima@fmu.ac.jp (M.O.); yasumura@fmu.ac.jp (S.Y.); 2 Sendai City Mental Health and Welfare Center, Sendai 980-0845, Japan

**Keywords:** posttraumatic growth, Fukushima evacuees, Fukushima nuclear accident, recovery from radiation anxiety

## Abstract

We examined the differences in the posttraumatic growth (PTG) free descriptions from clusters of Fukushima residents (evacuation and non-evacuation zones) who experienced the Great East Japan Earthquake, and the relationship between “recovery from radiation anxiety” and the PTG-free description classification in these regions. A mail survey was conducted in August 2016 among Fukushima residents aged 20–79 years for free descriptions of their PTG. Participants were then divided into the “no anxiety,” “recovered from anxiety,” and “unrecovered from anxiety” groups based on their “recovery from radiation anxiety.” Data from 786 responses were analyzed. The PTG-free descriptions were classified into eight categories. Among those who lived in the evacuation zone versus those in the non-evacuation zone, “relating to others” (non-evacuation zone: 11.9% vs. evacuation zone: 18.4%) and “appreciation of life” (non-evacuation zone: 2.7% vs. evacuation zone: 9.8%) were significantly higher, and “increased awareness of disaster prevention” (non-evacuation zone: 20.4% vs. evacuation zone: 8.0%) was significantly lower. In the evacuation zone, “renewed recognition of nuclear issues” was significantly lower than the expected value in the no anxiety group (3.1%) and significantly higher than the expected value in the recovered group (22.9%). Further studies are needed to build support measures and potentially aid in preparing for future disasters.

## 1. Introduction

A massive earthquake and tsunami occurred along the broader of the Pacific coast of eastern Japan on 11 March 2011 (the Great East Japan Earthquake; GEJE) that caused the Fukushima Daiichi nuclear power station accident [1]. Owing to the disaster, it has been crucial to acquire evidence to support Fukushima residents with mental and physical health problems and future disaster victims urgently and consistently [2,3,4,5]. In particular, Nakayama et al. [6] conducted a questionnaire survey (“Health and Information Survey”) to examine the relationship between information on life and health and the health status of the residents of the Fukushima Prefecture who experienced the GEJE. They aimed to report basic information that would contribute to the response to large-scale and nuclear disasters.

Life-threatening stressful events, such as disasters, have a considerable negative impact on those who experience them, causing mental health problems such as post-traumatic stress disorder [7]. Additionally, in recent years, it has been reported that those who have experienced such difficult events undergo positive psychological transformation known as posttraumatic growth (PTG) [8,9]. PTG refers to positive psychological change resulting from a struggle with a major life crisis or traumatic events [8,9]. Previous studies have reported evidence of the Posttraumatic Growth Inventory (PTGI) in various situations involving motor vehicle accidents [10], natural disasters [11], life-threatening diseases [12], war veterans [13], and assault [14].

Previous studies have reported a close relationship between PTG and resilience [15,16,17], which here describes “a stress-coping ability in the face of adversity, in other words, a psychological resource” [17]. The prior findings suggest that individuals who are more likely to experience PTG when encountering difficulties are prone to undergoing psychological re-adaptation through the recovery process of traumatic events. Thus, examining and identifying the current state of Fukushima residents’ PTG and related factors will aid in the provision of the necessary assistance they require. Additionally, it will provide scientific evidence for developing mental health support strategies for future disaster victims.

The present study draws on and expands the survey designed and conducted by Nakayama et al. [6]. A full description of the survey and associated methods is available in previous studies [14,18,19,20]. The major highlights relevant to the present study are summarized as follows. Iwasa et al. [18] analyzed the frequency of PTG occurrence, and the relationship between PTG occurrence and recovery from anxiety regarding the effects of radiation on participants’ health owing to the nuclear disaster (“recovery from anxiety”). The results showed that about 55% of the total experienced PTG, and those who recovered from anxiety about radiation health effects were more likely to experience PTG. In addition, Iwasa et al. [19] also examined in more detail what kind of PTG is likely to be reported, and whether the content of PTG differs depending on the basic attributes (i.e., age, gender, education) and the situation of recovery from radiation anxiety, using a free description of the specific content of the PTG measured by Nakayama et al. [6]. However, the actual conditions of the PTG descriptions in the residents of the evacuation zone who were directly affected by the impact of the huge earthquake have not yet been clarified.

Therefore, considering Fukushima residents who lived in the evacuation and non-evacuation zones, the present study examined (1) the relationship between these residential areas and the free description classification of PTG, and (2) the relationship between recovery from anxiety and the free description classification of PTG. Our findings will lead to the establishment of a framework for supporting the mental health of future victims, in addition to Fukushima residents.

## 2. Materials and Methods

### 2.1. Participants

The survey targeted 2000 Fukushima residents between the ages of 20 and 79 years. The Fukushima Prefecture is divided into four areas based on the general regional classification of Aizu, Nakadōri, Hamadōri, and the evacuation areas (including the restricted, evacuation-prepared, and deliberate evacuation areas as determined on 22 April 2011). For this study, 500 inhabitants were selected from each area. The selection was based on a two-step stratified random sampling. Nakadōri and Hamadōri included local governments that were partially in evacuation areas. The survey was conducted from August 15 to 17 October 2016, as an anonymous postal self-reported questionnaire (called the “Health and Information Survey”) [6]. The questionnaires returned were considered as confirmation that the participants agreed with the purpose of the survey and were willing to participate voluntarily. The present study was approved by the Fukushima Medical University Ethics Committee (approval number: 2699).

### 2.2. Measurements

#### 2.2.1. PTG

The item, “It goes without saying that the GEJE was a big negative experience; however, have you benefited in any way through the experience?” was used to evaluate PTG. The response options for this item were “yes/no,” and participants were asked to describe their experiences if they selected “yes”.

#### 2.2.2. Radiation Anxiety

For two different points in time—(1) at the time of the disaster (i.e., March 2011) and (2) at the time of the survey—participants were asked to rate the level of their anxiety related to the effects of radiation owing to the nuclear disaster on their health on a 5-point Likert-type scale (1 = “None,” through 5 = “Extreme”). Their responses were dichotomized as “no” (answers 1–3) or “yes” (answers 4 and 5) [20]. Furthermore, both the anxiety measures were combined to create a new item, “recovery from anxiety,” with three values (1 = “no anxiety” [“no” at the time of the disaster and survey], 2 = “recovery” [“yes” at the time of the disaster and “no” at the time of the survey], and 3 = “unrecovered” [“yes” at the time of the disaster and survey]) [18]. Based on the three values, the participants were categorized into three groups (i.e., “no anxiety,” “recovery,” and “unrecovered”). Participants who responded “no” for at the time of the disaster and “yes” for at the time of the survey were excluded from the analyses, since there were only a few of them (n = 3).

#### 2.2.3. Other Variables

To illustrate participants’ characteristics, the following data were collected: age, gender, education (dichotomized: elementary/secondary school or vocational school/university), living arrangements (living alone or with others), employment status (employed or unemployed), physical activity, alcohol consumption, smoking, self-rated health (dichotomized: excellent/very good/good or fair/poor), and the area where the participants lived at the time of the disaster (dichotomized: evacuation zone or non-evacuation zone).

### 2.3. Analytical Plans

#### 2.3.1. Participants’ Baseline Characteristics

To examine differences in baseline characteristic factors between the residential areas, we performed a chi-squared test for dichotomous variables and a t-test for continuous variables.

#### 2.3.2. PTG Free Description Classification

The PTG free description was classified, and the frequency (%) of each category was calculated. The present study followed the PTG free description classification created in our previous study [19]. We used classifications based on five dimensions of PTGI [8]: “relating to others,” “new possibilities,” “personal strength,” “spiritual change,” and “appreciation of life.” However, the following two points were classified according to previous studies. (1) The fourth dimension, “spiritual change,” of PTGI is said to be difficult for Japanese to extract owing to religious and cultural differences between Japan and European countries [21]. Nishino et al. [21] translated religious beliefs included in “spiritual change” into “feelings of belief in something beyond human power,” and created and investigated a modified PTGI scale for the Japanese people. In addition, Nishino et al. [22] included “impermanence” and “wonder and awe for nature” in the reaction in this area, in addition to the changes in religious activity after the traumatic experience. (2) As subcategories of PTGI’s second dimension “new possibilities,” considering the findings of Nishino et al. [22]: IIa, “expanding the field of view “ (transformation of values), IIb, “increased awareness of disaster prevention” (increased awareness of preparation for future disasters), IIc, “renewed recognition of nuclear power issues” (increasing interest in nuclear power issues and future energy policies), and IId, “critical examination of information from authorities” (heightened critical awareness of information transmitted by administrative, electric power companies, national newspapers, and national television broadcasts).

Based on the above criteria, the free descriptions were classified. First, the first author classified PTG free descriptions into eight categories based on the above criteria. Next, we asked one research collaborator (a part-time staff member of the department to which the first author belongs with no research experience, and no prior knowledge about this research theme) to confirm the classification results. If the two classifications were different, the final classification was decided through discussions between these two people. The degree of agreement (kappa coefficient) between the two was 0.947 (p <0.01). If it can be judged that the description has the same meaning, even if the description spans multiple sentences, it is counted as one. We analyzed 503 descriptions, excluding 15 descriptions that deviate from the definition of PTG, such as “I am worried about changes in life owing to the earthquake.”

#### 2.3.3. Assessing the Relationship between PTG Free Description Classification and Residential Areas

A cross-tabulation and a chi-squared test were performed to examine the relationship between PTG free description classification and residential areas.

#### 2.3.4. Assessing the Relationship between PTG Free Description Classification and Recovery from Anxiety by Residential Areas

A cross-tabulation and a chi-square test were performed to examine the relationship between PTG free description classification and recovery from anxiety by residential areas. If significant, residual analyses were performed to identify cells with significantly higher (lower) frequencies than the expected values.

The significance level in all statistical tests was set at 5%.

## 3. Results

We received 916 responses to the 1985 questionnaires that were initially distributed (excluding those that were returned to us because no one was currently residing at the address) [6]. Of these, 127 questionnaires were excluded from the analyses for the following reasons: 55 had left the age or gender column blank, and 53, 9, and 10 questionnaires omitted values for PTG, radiation anxiety, and education attainment items, respectively. In addition, three questionnaires of individuals who answered “no anxiety” at the time of the disaster and “anxiety” at the time of the survey were excluded from all analyses (see Section 2.2.2). Consequently, data from 786 individuals were used for the final analyses. Table 1 shows the participants’ characteristics.

### 3.1. Frequencies of Categories for the PTG Free Description

Of the 436 people with PTG marked “Yes”, 424 responded with free descriptions. Of the 503 descriptions of meaning and content, the largest number was IIb, “increased awareness of disaster prevention”, at 140 (27.8%), followed by I, “relationship with others”, at 104 (20.1%), and IIc, “renewed recognition of nuclear issues”, was at 77 (15.3%). Table 2 shows examples of free descriptions according to PTG free description categories.

### 3.2. Comparison of Categories of PTG Free Descriptions According to Regions Where Participants Lived When the Disaster Occurred (Non-Evacuation vs. Evacuation Zone)

The proportion of those who answered I, “relating to others”, (non-evacuation zone: 11.9% vs. evacuation zone: 18.4%; chi-square = 4.79, df = 1, *p* = 0.029) and V, “appreciation of life”, (non-evacuation zone: 2.7% vs. evacuation zone: 9.8%; chi-square = 16.13, df = 1, *p* < 0.001) were significantly higher among participants who lived in the evacuation zone. Meanwhile, the proportion of those who answered IIb, “increased awareness of disaster prevention”, (non-evacuation zone: 20.4% vs. evacuation zone: 8.0%; 8.0%; chi-square = 13.59, df = 1, *p* < 0.001) was significantly lower in participants who lived in the evacuation zone (Table 3).

### 3.3. Associations of Recovery from Anxiety with Categories of PTG Free Descriptions: Comparison of Non-Evacuation and Evacuation Zone

Among those who lived in the non-evacuation zone, the proportion of those who answered III, “personal strength”, (1.5%; chi-square = 8.84, df = 2, *p* = 0.017), and IV, “spiritual change”, (2.7%; chi-square = 10.13, df = 2, *p* = 0.006) were significantly lower than the expected values in the no anxiety group. The proportion of those who answered III, “personal strength”, (6.1%; chi-square = 8.84, df = 2, *p* = 0.012) was higher than the expected value in the recovery group. The proportion of those who answered IIb, “increased awareness of disaster prevention”, (8.8%; chi-square = 7.08, df = 2, *p* = 0.029) was significantly lower than the expected value in the unrecovered group. Finally, the proportion of those who answered IV, “spiritual change”, (10.3%; chi-square = 10.14, df = 2, *p* = 0.006) was significantly higher than the expected value in the unrecovered group (Table 4).

Among those who lived in the evacuation zone, the proportion of those who answered IIc, “renewed recognition of nuclear issues”, was significantly lower than the expected value in the no anxiety group (3.1%; chi-square = 12.97, df = 2, *p* = 0.002). The proportion of those who answered IIc, “renewed recognition of nuclear problems”, was significantly higher than the expected value in the recovered group (22.9%; chi-square = 12.97, df = 2, *p* = 0.002) (Table 4).

## 4. Discussion

The present study aimed to examine (1) the relationship between residential areas and the free description classification, and (2) the relationship between recovery from anxiety and the free description classification of PTG for Fukushima residents who lived in the evacuation zone, compared with those in the non-evacuation zone. The proportion of those who answered “relating to others” and “appreciation of life” was significantly higher among those who lived in the evacuation zone than those in the non-evacuation zone. The proportion of those who answered “increased awareness of disaster prevention” was significantly lower in the persons who lived in the evacuation zone than those in the non-evacuation zone. Among persons who lived in the evacuation zone, the proportion of those who answered “renewed recognition of nuclear problems” was significantly lower than the expected value in the no anxiety group, and the proportion was significantly higher than the expected value in the recovered group.

### 4.1. Comparison of PTG Free Descriptions Classification between Non-Evacuation and Evacuation Zone

In the evacuation zone, where the effects of the tsunami and evacuation were immense, it is likely that many people cited I, “relating to others”, as PTG, because, through the prolonged evacuation, they received the generous support of local governments and volunteers. The word “KIZUNA,” which means “bond” (e.g., bond with people, and bond to a community), was also popular in Japan in 2011 [23].

Since the evacuees faced great damage caused by the disaster, and now, difficulty returning to their hometowns, they were likely to be keenly aware of the gratitude for their lives before the earthquake, and to report V, “appreciation of life”, as PTG. A previous study [22] also reported a narration about appreciations for normal daily life (before the earthquake) as follows, “I learned the value of normal life: fleeing thankful for being able to use electricity, having water, taking a warm bath, and sleeping on a futon.”

Among those who lived in the evacuation zone, the proportion of those who answered IIb, “increased awareness of disaster prevention”, was low. This is because, in the evacuation zone where the damage was severe, reconstruction is still underway, and it seems that awareness of preparing for future disasters is still low. Contrarily, the residents of the non-evacuation zone received relatively less damage and witnessed the damage in the prefecture through the media. Thus, they could be more likely to respond to IIb, “increased awareness of disaster prevention”, as PTG to prepare for future disasters.

### 4.2. Relationship between Recovery from Anxiety and PTG Free Description Classification: Examination by Residential Area

Among participants who lived in the evacuation zone, the proportion of those who reported IIc, “renewed recognition of nuclear issues”, was low in the no anxiety group and high in the recovery group. The recovery group recovered after exposure to radiation anxiety. In the process, their attention has been focused from the past to the future, and new values may have been created with the aim of realizing a safer and more sustainable society. A previous study [22] also reported a narration as follows, “I feel the need to pay close attention to nuclear power plants in the future. Now more than ever, I would like to participate in the movement against them and the movement supporting the introduction of renewable energy,” which reported that new goals were born from experience, and new hopes were born from thoughts for reconstruction. In light of this, the present study, cited a similar free description in IIc, “renewed recognition of nuclear issues”, to represent new goals and hopes found in the recovered from anxiety group.

Among those living in the non-evacuation zone, in the unrecovered group, the percentage of those who reported IIb, “awareness of disaster prevention”, was low. The unrecovered group was exposed to radiation anxiety at the time of the disaster, and the anxiety persisted even during the survey. It is presumed that return to normalcy has not been achieved in the living conditions and mental health of the group. With such strong attention being paid to the current anxiety, it is unlikely that future prospects, such as preparation for future disaster prevention, will occur.

Additionally, the percentage of those who reported IV, “spiritual change”, was high in the unrecovered group. The unrecovered group was expected to be in the midst of the struggle with a major life crisis or traumatic events that produce PTG [8], and it is thought that such an internal situation would naturally be reflected in the PTG. We felt that the unstable mental state of the group may have resulted in “reality of death and back-to-back” and the sense of “importance of life” that emerged from such extreme situations, as well as “impermanence” and “the awe of natural disasters and nuclear power plants which were beyond human power.”

### 4.3. Limitations of the Study

The present study had several limitations. First, although the participation proportion in the present study was the same as that of similar surveys [24], it was not high. Therefore, the representativeness of our sample may have been limited. Therefore, caution should be exercised when generalizing the findings. Second, because we conducted a cross-sectional survey, causal relationships could not be inferred. Regarding the relationship between recovery from anxiety and PTG, the present study could not clarify whether recovery from anxiety causes PTG, or vice versa. Future research using a longitudinal study design is needed to reveal the cause–effect relationship. Third, in the present study, because the sample size was not sufficient and multivariate analysis was not performed, confounding factors were not controlled in the following: the relationship between residential area and PTG free description classification; and the relationship between recovery from anxiety and PTG free description classification. In a future study, conducting multivariate analyses of the relationships would be required to examine the independent relationships. Fourth, PTG was measured for one item, and a free description was requested in addition. This was to identify and examine the emotions and PTG of Fukushima residents. It is undeniable that by adding “It goes without saying that the GEJE is a big negative experience,” to the question text, more positive descriptions from participants could have increased. Fifth, regarding the validity of the PTG free description classification, in the present study, PTG was measured for a single item with a free description of participants’ experiences. Subsequently, the obtained free descriptions were classified with reference to the findings of previous studies [8,19,22]. Furthermore, we conducted a quantification of such qualitative responses and examined the differences in the PTG free description classification between residential areas. We further assessed the relationship between the PTG free description classification and recovery from anxiety. As mentioned above, the validity of the PTG free description classification may not be sufficiently guaranteed. Specifically, the newly created categories in the present study (categories IIb-IId) were insufficient to verify whether it conforms to the existing PTG concept. From the above issues, caution should be applied regarding the generalization of the present findings. Finally, the validity and reproducibility of the measurement for radiation anxiety has not yet been verified. However, Nakayama et al. [6], who used the same items, found relationships between low levels of radiation anxiety and confidence in information on radiation (e.g., believing in information released by the government), high self-rated health, employment status, and high level of health literacy [25]. Although the validity of the cut-off value for radiation anxiety has also not yet been verified, this standard was based on a previous study [20].

## 5. Conclusions

The present study provides valuable information for understanding the psychological characteristics of Fukushima residents (especially those who lived in evacuation areas) who experienced the GEJE by investigating their actual PTG conditions. For victims of huge disasters such as the GEJE, it is possible that there are relatively large individual differences in reconstruction not only in terms of living conditions, but also in terms of mental conditions. A possibility is that residents of the evacuation zone, who are thought to be more affected by the disaster, valued “bonds” with their families, friends, and the community (i.e., KIZUNA in Japanese, as cited above) greatly, and strongly appreciated their daily lives, which helped them recover from the disaster. Furthermore, during psychological re-adaptation, while reconstructing their society, a renewed recognition of nuclear issues may have sprung up. Considering the psychological characteristics of the residents in the evacuation zone, further studies are required to build support measures for the mental health of the victims, and potentially aid in preparing for future disasters.

## Figures and Tables

**Table 1:** Distribution of participants’ characteristics.

	Non-Evacuation Zone (n = 623)	Evacuation Zone (n = 163)	*p*
Age, mean (SD)	55.71 (14.89)	57.51 (15.05)	0.168
Gender (women), n (%)	331 (53.1)	92 (56.4)	0.450
Primary education, n (%)	76 (12.2)	30 (18.4)	0.050
Secondary education, n (%)	319 (51.2)	86 (52.8)	
Higher education, n (%)	228 (36.6)	47 (28.8)	
Living alone, n (%)	71 (11.4)	25 (15.4)	0.165
Employment (employed), n (%)	403 (65.2)	62 (39.0)	<0.001
Physical activity (yes), n (%)	324 (52.6)	93 (57.1)	0.310
Drinking, n (%)	179 (29.0)	52 (31.9)	0.472
Smoking, n (%)	122 (19.7)	37 (22.8)	0.384
Self-rated heath (good), n (%)	314 (50.7)	58 (35.6)	<0.001
Radiation anxiety at the time of the disaster, n (%)	216 (34.7)	65 (39.9)	0.217
Radiation anxiety at present, n (%)	68 (10.9)	30 (18.4)	0.010
Recovery from anxiety			
No anxiety, n (%)	407 (65.3)	98 (60.1)	0.036
Recovered from anxiety, n (%)	148 (23.8)	35 (21.5)	
Unrecovered from anxiety, n (%)	68 (10.9)	30 (18.4)	
PTG occurrence, n (%)	345 (55.4)	91 (55.8)	0.918

PTG = posttraumatic growth. To examine differences in baseline characteristic factors between the residential areas, we performed a chi-squared test for dichotomous variables and a *t*-test for continuous variables.

**Table 2:** Categories for PTG free descriptions.

Categories	Summary	Examples of Free Descriptions
I. Relating to others	Realizing the relationships with my family and friends, and the ties with the community	- “Children were more worried than I expected and I felt the gratitude of my family.” - “I felt that the negative experience strengthened the bonds in the region.”
IIa. Expanding the field of view	Transformation of values that causes new behavior (i.e., new goals have been found, altruism has sprung up, and people’s perspectives have changed)	- “The breadth of life has expanded.” - “I could clearly see who was warm-hearted and who was cold-hearted.”
IIb. Increased awareness of disaster prevention	Crisis management, increased awareness of disaster prevention, the importance of resources such as water, electricity, gas, and food, and re-evaluation of food safety have occurred.	- “Because I don’t know when a disaster will occur, I started to keep all my regular items together, especially after the earthquake.” - “I will judge by looking at the difference between the information provided by the country or university and the national newspapers and books.”
IIc. Renewed recognition of nuclear issues	Re-recognition of nuclear power (including radioactive materials) and energy issues has occurred.	- “Awareness of safety for nuclear power plants has changed.” - “I received knowledge of radiation, and experienced fear for the nuclear power and the nuclear power plant.”
IId. Critical examination for information from authorities	Information issued by the government (country/prefecture), electric power companies, national newspapers, and national television broadcasts is now critically examined without being blindly accepted.	- “Information on countries and experts (in Japan) cannot be trusted.” - “Commercial TV broadcasts dare to raise anxiety (for instance, they make general consumers say that they are worried because they have small children at home).”
III. Human strength	Recognizing one’s strength (including flexibility, generosity, mental toughness)	- “I realized strength to overcome painful things.” - “I came to collect information by myself and decide what action to take next.”
IV. Spiritual change	Experiencing spiritual and religious growth (including awe of nature and human powerlessness, awareness of life and death, impermanence)	- “I learned and realized that I should not underestimate nature.” - “I experienced reality of death and back to back.”
V. Appreciation of life	Gratitude for the life I took for granted and for my life so far	- “I am thankful for being able to live a normal life.” - “It was natural to live in a place where I used to live, but now it’s irreplaceable hometown to me.”

**Table 3:** Associations between the residential areas and the categories for PTG free descriptions.

Categories	Non-Evacuation Zone (n = 623)	Evacuation Zone (n = 163)	*P* *
I. Relating to others	74 (11.9)	30 (18.4)	0.029
IIa. Expanding the field of view	33 (5.3)	9 (5.5)	0.910
IIb. Increased awareness of disaster prevention	127 (20.4)	13 (8.0)	<0.001
IIc. Renewed recognition of nuclear issues	63 (10.1)	14 (8.6)	0.560
IId. Critical examination for information from authorities	39 (6.3)	6 (3.7)	0.207
III. Human strength	18 (2.9)	7 (4.3)	0.363
IV. Spiritual change	28 (4.5)	9 (5.5)	0.581
V. Appreciation of life	17 (2.7)	16 (9.8)	<0.001

* A chi-square test was conducted.

**Table 4:** Associations between “recovery from radiation anxiety” and the categories of PTG free descriptions.

	Non-Evacuation Zone (n=623)				Evacuation Zone (n = 163)			
Categories	No Anxiety (n = 407)	Recovered from Anxiety (n = 148)	Unrecovered from Anxiety (n = 68)	*p* *	No Anxiety (n = 98)	Recovered from Anxiety (n = 35)	Unrecovered from Anxiety (n = 30)	*p* *
I. Relating to others	43 (10.6)	25 (17.6)	6 (8.1)	ns	18 (18.4)	7 (20.0)	5 (16.7)	ns
IIa. Expanding the field of view	25 (6.1)	6 (4.1)	2 (2.9)	ns	4 (4.1)	4 (11.4)	1 (3.3)	ns
IIb. Increased awareness of disaster prevention	85 (20.9)	36 (24.3)	6 (8.8)↓	0.029	8 (8.2)	3 (8.6)	2 (6.7)	ns
IIc. Renewed recognition of nuclear issues	35 (8.6)	17 (11.5)	8 (11.8)	ns	3 (3.1)↓	8 (22.9)↑	3 (10.0)	<0.001
IId. Critical examination for information from authorities	22 (5.4)	14 (9.5)	6 (8.8)	ns	3 (3.1)	3 (8.6)	0 (0.0)	ns
III. Human strength	6 (1.5)↓	9 (6.1)↑	3 (4.4)	0.012	6 (6.1)	1 (2.9)	0 (0.0)	ns
IV. Spiritual change	11 (2.7)↓	10 (6.8)	7 (10.3)↑	0.006	6 (6.1)	1 (2.9)	2 (6.7)	ns
V. Appreciation of life	12 (2.9)	3 (2.0)	2 (2.9)	ns	9 (9.2)	4 (11.4)	3 (10.0)	ns

↑: Residual analysis showed that the number of people in the cell was statistically larger than the expected value (*p* < 0.05). ↓: Residual analysis showed that the number of people in the cell was statistically smaller than the expected value (*p* < 0.05). * A chi-square test was conducted.

## Data Availability

Data is not suitable for public deposition owing to ethical concerns. Researchers who have an interest in the analysis using the data, please submit requests to the Fukushima Medical University Ethics Committee (rs@fmu.ac.jp) for access to confidential data.

## References

[1] Yasumura S., Hosoya M., Yamashita S., Kamiya K., Abe M., Akashi M., Kodama K., Ozasa K. (2012). Study protocol for the Fukushima Health Management Survey. J. Epidemiol..

[2] Yabe H., Suzuki Y., Mashiko H., Nakayama Y., Hisata M., Niwa S., Yasumura S., Yamashita S., Kamiya K., Abe M. (2014). Psychological distress after the Great East Japan Earthquake and Fukushima Daiichi Nuclear Power Plant accident: Results of a mental health and lifestyle survey through the Fukushima Health Management Survey in FY2011 and FY2012. Fukushima J. Med. Sci..

[3] Hasegawa A., Tanigawa K., Ohtsuru A., Yabe H., Maeda M., Shigemura J., Ohira T., Tominaga T., Akashi M., Hirohashi N. (2015). Health effects of radiation and other health problems in the aftermath of nuclear accidents, with an emphasis on Fukushima. Lancet.

[4] Bromet E.J. (2012). Mental health consequences of the Chernobyl disaster. J. Radiol. Prot..

[5] Horikoshi N., Maeda M., Iwasa H., Momoi M., Oikawa Y., Ueda Y., Kashiwazaki Y., Onji M., Harigane M., Yabe H. (2020). The Usefulness of brief telephonic intervention after a nuclear crisis: Long-term community-based support for Fukushima evacuees. Disaster Med. Public Health Prep..

[6] Nakayama C., Sato O., Sugita M., Nakayama T., Kuroda Y., Orui M., Iwasa H., Yasumura S., Rudd R.E. (2019). Lingering health-related anxiety about radiation among Fukushima residents as correlated with media information following the accident at Fukushima Daiichi Nuclear Power Plant. PLoS ONE.

[7] Fujii S., Oe M., Maeda M. (2014). A review of Long-term effects of man-made disasters and natural disasters. Jpn. J. Trauma. Stress.

[8] Tedeschi R.G., Calhoun L.G. (1996). The Posttraumatic Growth Inventory: Measuring the positive legacy of trauma. J. Trauma. Stress.

[9] Iimura S. (2016). Perceptions toward posttraumatic growth: Life crisis and positive psychological changes. Stress Manag. Res..

[10] Zoellner T., Rabe S., Karl A., Maercker A. (2008). Posttraumatic growth in accident survivors: Openness and optimism as predictors of its constructive or illusory sides. J. Clin. Psychol..

[11] Xu J., Liao Q. (2011). Prevalence and predictors of posttraumatic growth among adult survivors one year following 2008 Sichuan earthquake. J. Affect. Disord..

[12] Teixeira R.J., Pereira M.G. (2013). Factors contributing to posttraumatic growth and its buffering effect in adult children of cancer patients undergoing treatment. J. Psychosoc. Oncol..

[13] Tsai J., El-Gabalawy R., Sledge W.H., Southwick S.M., Pietrzak R.H. (2015). Post-traumatic growth among veterans in the USA: Results from the National Health and Resilience in Veterans Study. Psychol. Med..

[14] Shakespeare-Finch J., de Dassel T. (2009). Exploring posttraumatic outcomes as a function of childhood sexual abuse. J. Child Sex. Abus..

[15] Kong L., Fang M., Ma T., Li G., Yang F., Meng Q., Li Y., Li P. (2018). Positive affect mediates the relationships between resilience, social support and posttraumatic growth of women with infertility. Psychol. Health Med..

[16] Liu Y., Li Y., Chen L., Li Y., Qi W., Yu L. (2018). Relationships between family resilience and posttraumatic growth in breast cancer survivors and caregiver burden. Psycho-Oncology.

[17] Nishi D., Kawashima Y., Noguchi H., Usuki M., Yamashita A., Koido Y., Matsuoka Y.J. (2016). Resilience, post-traumatic growth, and work engagement among health care professionals after the Great East Japan Earthquake: A 4-year prospective follow-up study. J. Occup. Health.

[18] Iwasa H., Moriyama N., Kuroda Y., Nakayama C., Orui M., Horiuchi T., Nakayama T., Sugita M., Yasumura S. (2019). Recovery from radietion anxiety and posttraumatic growth among community dwellers after the nuclear disaster in Fukushima. Cogent. Psychol..

[19] Iwasa H., Nakayama C., Moriyama N., Orui M., Yasumura S. (2021). Posttraumatic growth after the Fukushima nuclear disaster: Examination of free descriptions among Fukushima residents. Nihon Koshu Eisei Zasshi (Jpn. J. Public Health).

[20] Orui M., Nakayama C., Kuroda Y., Moriyama N., Iwasa H., Horiuchi T., Nakayama T., Sugita M., Yasumura S. (2020). The Association Between Utilization of Media Information and Current Health Anxiety Among the Fukushima Daiichi Nuclear Disaster Evacuees. Int. J. Environ. Res. Public Health.

[21] Nishino A., Sawazaki T. (2014). A study of posttraumatic growth through adversity-Focus on the subjective meaning of one’s experience. Mejiro J. Psychol..

[22] Nishino M., Ito T. (2013). A text mining study of college students’ narratives after the 3.11 Disaster. Bull. Tohoku Fukushi Univ. Grad. Sch..

[23] The Japan Kanji Aptitude Testing Foundation The First Place in “Kanji of the Year 2011”: “KIZUNA”. https://www.kanken.or.jp/project/edification/years_kanji/2011.html.

[24] Fukasawa M., Kawakami N., Umeda M., Miyamoto K., Akiyama T., Horikoshi N., Yasumura S., Yabe H., Bromet E.J. (2017). Environmental radiation level, radiation anxiety, and psychological distress of non-evacuee residents in Fukushima five years after the Great East Japan Earthquake: Multilevel analyses. SSM Popul. Health.

[25] Ishikawa H., Nomura K., Sato M., Yano E. (2008). Developing a measure of communicative and critical health literacy: A pilot study of Japanese office workers. Health Promot. Int..

